# Validity Evidence Based on Relations to Other Variables of the eHealth Literacy Questionnaire (eHLQ): Bayesian Approach to Test for Known-Groups Validity

**DOI:** 10.2196/30243

**Published:** 2021-10-14

**Authors:** Christina Cheng, Gerald Elsworth, Richard H Osborne

**Affiliations:** 1 Centre for Global Health and Equity School of Health Sciences Swinburne University of Technology Hawthorn Australia; 2 School of Health and Social Development Faculty of Health Deakin University Burwood Australia

**Keywords:** eHealth, digital health, health literacy, health equity, questionnaire design, health literacy questionnaire, validity evidence, mediation effect, mobile phone

## Abstract

**Background:**

As health resources and services are increasingly delivered through digital platforms, eHealth literacy is becoming a set of essential capabilities to improve consumer health in the digital era. To understand eHealth literacy needs, a meaningful measure is required. Strong initial evidence for the reliability and construct validity of inferences drawn from the eHealth Literacy Questionnaire (eHLQ) was obtained during its development in Denmark, but validity testing for varying purposes is an ongoing and cumulative process.

**Objective:**

This study aims to examine validity evidence based on relations to other variables—using data collected with the known-groups approach—to further explore if the eHLQ is a robust tool to understand eHealth literacy needs in different contexts. A priori hypotheses are set for the expected score differences among age, sex, education, and information and communication technology (ICT) use for each of the 7 eHealth literacy constructs represented by the 7 eHLQ scales.

**Methods:**

A Bayesian mediated multiple indicators multiple causes model approach was used to simultaneously identify group differences and test measurement invariance through differential item functioning across the groups, with ICT use as a mediator. A sample size of 500 participants was estimated. Data were collected at 3 diverse health sites in Australia.

**Results:**

Responses from 525 participants were included for analysis. Being older was significantly related to lower scores in 4 eHLQ scales, with *3. Ability to actively engage with digital services* having the strongest effect (total effect –0.37; P<.001), followed by *1. Using technology to process health information* (total effect –0.32; P<.001), *5. Motivated to engage with digital services* (total effect –0.21; P=.01), and *7. Digital services that suit individual needs* (total effect –0.21; P=.02). However, the effects were only partially mediated by ICT use. Higher education was associated with higher scores in *1. Using technology to process health information* (total effect 0.22; P=.01) and *3. Ability to actively engage with digital services* (total effect 0.25; P<.001), with the effects mostly mediated by ICT use. Higher ICT use was related to higher scores in all scales except *2. Understanding health concepts and language* and *4. Feel safe and in control*. Either no or ignorable cases of differential item functioning were found across the 4 groups.

**Conclusions:**

By using a Bayesian mediated multiple indicators multiple causes model, this study provides supportive validity evidence for the eHLQ based on relations to other variables as well as established evidence regarding internal structure related to measurement invariance across the groups for the 7 scales in the Australian community health context. This study also demonstrates that the eHLQ can be used to gain valuable insights into people’s eHealth literacy needs to help optimize access and use of digital health and promote health equity.

## Introduction

### Background

eHealth literacy, also known as digital health literacy, has been described as a set of essential capabilities to improve consumer health in the digital era [1,2]. As health resources and services continue to move to digital platforms, people need “the ability to seek, find, understand, and appraise health information from electronic sources and apply the knowledge gained to addressing or solving a health problem” [1]. Digital health will have limited value if people do not have adequate eHealth literacy to effectively engage with these resources [1,3,4]. Hence, an understanding of eHealth literacy needs is paramount to ensure that digital health resources are aligned with such needs and avoid the potential widening of health inequities. However, current research and insights into the eHealth literacy needs of populations are limited, and the results can be inconsistent [5], possibly because of the lack of a rigorous theoretical framework to measure eHealth literacy [6].

To understand eHealth literacy needs, a useful and valid measurement of eHealth literacy is needed. With the introduction of this concept in 2006, the study by Norman and Skinner [7] developed the eHealth Literacy Scale (eHEALS) to assess people’s ability to engage with eHealth, with the purpose of informing clinical decisions and health promotion planning. Initial validity testing of the tool in Canada demonstrated good internal consistency (coefficient α=.88), with principal component analysis suggesting a single-factor solution [7]. However, recent validation studies have cast doubts on the tool’s dimensionality. The tool was found to be a better fit for a two-factor model in 5 studies, but there was no consensus on the items for the 2 subscales among these studies [8-12], whereas 3 studies reported that the eHEALS consisted of 3 dimensions [13-15]. Nevertheless, the tool has been widely used in eHealth literacy studies across the world in various settings to understand the eHealth literacy of various population groups, examine the association of eHealth literacy and sociodemographic factors, measure the effects of eHealth literacy on health outcomes, and use as an outcome measure of eHealth literacy interventions [6,16]. Yet, the linkages of the findings of these studies to specific eHealth recommendations were usually vague [6]. In contrast, the study by Norman [17] acknowledged that the digital landscape had evolved since 2006, especially around the interactivity and expanded capabilities of information and communication technologies (ICTs), and called for revision of the concept as well as its measurement tool.

Using a grounded validity-driven approach [18], the study by Norgaard et al [19] developed the eHealth Literacy Framework by integrating the perspectives and experiences of a wide range of eHealth stakeholders, including patients, health care providers, health informatics professionals, public health researchers, and computer scientists. Through concept-mapping workshops and international web-based surveys, 7 domains of eHealth literacy were identified [19]. On the basis of the eHealth Literacy Framework and on the back of the widely used and tested Health Literacy Questionnaire [20], the eHealth Literacy Questionnaire (eHLQ) was subsequently developed comprising 7 scales and representing the following 7 eHealth literacy constructs:

1. Using technology to process health information
2. Understanding of health concepts and language
3. Ability to actively engage with digital services
4. Feel safe and in control
5. Motivated to engage with digital services
6. Access to digital services that work
7. Digital services that suit individual needs [21]

Each eHLQ scale has 4 to 6 items relating to a 4-point ordinal scale, ranging from strongly disagree to strongly agree. The results are 7 scale scores with a range of 1-4, calculated by averaging the item scores within each scale with equal weighting. Initial validity testing of the eHLQ involved extensive discussion of the test content in the Australian and Danish contexts by an international multidisciplinary team experienced in questionnaire development and cognitive interviewing with community members from different cultural and educational backgrounds to ensure that the items were understood as intended. The items were then administered to 475 Danish participants randomly approached by trained interviewers in the broader-community locations, including libraries, workplaces, hospitals, nursing homes, health centers, and an outpatient clinic. Bayesian confirmatory factor analysis supported the seven-factor model, with all items loading strongly on their relevant factors and no statistically significant cross-loadings. Composite scale reliability (ranging from 0.75 to 0.87) demonstrated good internal consistency. Item response theory analysis confirmed that there were no disordered thresholds, and differential item functioning (DIF) testing established evidence of measurement invariance for age and sex [21]. The eHLQ has since been used to investigate the eHealth literacy of nursing students and pregnant migrant women in Denmark [22,23], as well as to examine the association of eHealth literacy and digital health service use in both Denmark and Australia [24,25]. The tool has also been used in Australia to understand the eHealth literacy needs of community members, leading to the generation of numerous concrete solutions to address the identified needs [26].

### Study Aim

According to the *Standards for Educational and Psychological Testing* (the *Standards*) [27], the authoritative reference used to develop, use, and interpret educational and psychological measurements, validity testing is a continuous process and involves the examination of 5 sources of evidence to support the interpretation and use of the scores, including test content, response process, internal structure, relations to other variables, and consequences of testing [27]. This study aims to examine evidence based on relations to other variables to further evaluate the eHLQ as a tool used to understand eHealth literacy needs.

Evidence of relations to other variables refers to an analysis of the relationship between the eHLQ scores and other variables with which the scores are predicted by theory or past research to be associated. The evidence may include association of the scores with certain demographic groups, relationships with predicted outcomes, or relationships between the scores and other external instruments that measure the same construct [27,28]. As there is no consensus on the dimensionality of the eHEALS, which is the most commonly used eHealth literacy tool, comparing the scores of the 2 instruments would be problematic. Therefore, this study focused on the testing of the association of the eHLQ scores with certain demographic groups, which is usually described as known-groups validity. A seminal paper on validity by Cronbach and Meehl [29] discussed that “If our understanding of a construct leads us to expect 2 groups to differ on the test, this expectation may be tested directly” [29]. Hence, group differences can be used to examine if an instrument is sensitive enough to discriminate “between these groups” [30]. However, the paper by Cronbach and Meehl [29] further noted that only moderate association should be expected because members of the groups were expected to overlap on the test, whereas failure to find a difference would also have serious implications for the test [29].

### Hypotheses Setting

#### Literature Review

To evaluate known-groups validity, hypotheses based on theoretical and empirical evidence need to be set up and tested. As this is an emergent field of research, studies on the predictors of eHealth literacy are limited, and inconsistent results are common [5]. Nevertheless, it has been argued that inequalities due to sociodemographic factors will affect the use of technology, acquisition of skills, and digital literacy. Conventional evidence, both theoretical and empirical, generally suggests that age, sex, education, and ICT use are associated with the ability to use technology for health, which will in turn potentially link to a person’s eHealth literacy [31-33]. Hence, a literature review was undertaken to generate hypotheses about the expected score differences across age, sex, education, and ICT use in relation to the 7 constructs or latent variables (ie, traits that cannot be directly observed or measured) representing the 7 scales of the eHLQ.

#### Age

People aged above 65 years are less likely than the younger generation to have had the chance to familiarize themselves with ICT either at school or at work [34,35]. Combined with the cognitive, motor, and sensory decline associated with aging, older adults face more barriers to and challenges in using technology for health than their younger counterparts [35-42]. With inadequate skill and ability, older people are more likely to experience computer anxiety [34,38], leading to less interest in using technology for health [34,38,43]. Slower processing of information and reduction of working memory caused by cognitive decline [39] can also lead to difficulty in understanding health concepts. In a systematic review of the use of digital health records among older adults, the 2 main barriers identified were privacy and security and access to, and ability to use, technology and the internet [42]. Hence, it was hypothesized as follows:

- H1: Age is negatively related to the scores on all latent variables representing the 7 scales.

#### Sex

Technology is traditionally perceived as a male-dominated domain, with men usually reporting higher levels of digital skills than women [32,44]. However, the study by Hargittai and Shafer [45] found no significant difference in the skill of web-based information searching by men and women in actual performance tests. Empirical findings indicated that women were more likely and more inclined to search for health information using the internet [38,46-48], with studies continuing to report that men tend to lag behind women in health knowledge [49-51]. The study by Brouwer et al [52] also found that women recorded a higher participation rate for a web-based health intervention than men, and women were more likely to engage in preventive activities related to health than men [49]. In terms of privacy concerns, no discussion of sex differences could be identified from the studies. These considerations led to the following two hypotheses:

- H2a: Being female is related to higher scores on the 3 latent variables representing the scales *1. Using technology to process health information, 2. Understanding of health concepts and language, and 5. Motivated to engage with digital services*.
- H2b: Sex is not related to score differences on the 4 latent variables representing the other 4 scales.

#### Education

Many studies have found education to be a predictor of ICT use and skills [32,34,35,38,53]. People with limited literacy, because of their limited ability to read and write, are likely to have less extensive health knowledge [49-51,54,55]. The generally higher-than-average reading level of web-based health information [56,57] may also disadvantage people with limited literacy. In addition, access to digital services to connect with health professionals generally requires some ability to read and write [58]. Besides, studies continue to find that searching for web-based health information, interpreting such information, and making decisions based on it is challenging for people with low literacy [36,59-61]. A further deterrence to using technology for health among people with limited literacy is that they tend to have greater privacy concerns because of mistrust of the internet and limited understanding of its capabilities [62]. Therefore, it was hypothesized as follows:

- H3: Education is positively related to the scores on all latent variables representing the 7 scales.

#### ICT Use

Higher ICT use is frequently found to be related to better digital skills and higher likelihood of searching for health information and using web-based health information and health apps [35,43,53,63]. With skills and access, people are more likely to be motivated to adopt and use web-based health resources with ease [34]. Furthermore, frequent use of ICT will also improve skills to deal with digital privacy concerns [62]. However, the relationship between ICT use and health knowledge has been hardly explored in the literature. These findings led to the following hypothesis:

- H4: ICT use is positively related to the scores on the 6 latent variables representing scales 1, 3, 4, 5, 6, and 7 but not to the score on the latent variable representing *2. Understanding of health concepts and language*.

#### Language

Considering that Australia is a multicultural country, whether the nation’s main language—English—was spoken at home was also included for analysis. However, because of the limited studies on the eHealth literacy of ethnic minorities [64] and because no studies could be identified targeting migrants who could speak the main language of their adopted country, no hypothesis was formulated for this group.

## Methods

### Data Collection

A cross-sectional survey was conducted across 3 health sites in Victoria, Australia, in 2018. The 3 sites included a private primary care medical clinic and a not-for-profit community health organization located in metropolitan areas as well as a private primary care medical clinic in a regional area. These sites were selected because they represented a mix of advantaged, disadvantaged, culturally diverse, metropolitan, and regional areas to ensure that the sample would capture people with different eHealth literacy levels. People attending the health sites were invited to participate if they were aged 18 years or older, with or without any health conditions, and were able to complete the eHLQ in paper-based format, web-based format, or face-to-face interview. The option of offering interviews allowed people with lower literacy who were not interested in reading to feel comfortable to participate in the survey, another strategy to ensure that people with potentially lower eHealth literacy were included. The exclusion criteria included people currently experiencing significant cognitive or mental health issues or too clinically unwell as deemed by their treating health care professionals and those with insufficient fluency in English to complete the survey because no family member or carer was present to assist them. The study was approved by the Deakin University Human Research Ethics Committee (approval number: HEAG-H 146_2017). Potential participants were provided information about the study, including that participation was voluntary. Returning the completed questionnaire was regarded as implied consent.

Demographic data collected for analysis included age, sex (male or female), education (less than secondary school, completed secondary school, certificate or diploma, or completed university or higher), language (spoke English at home or not), and ICT use. The classification of education into 4 categories was somewhat arbitrary. Other Australian studies of eHealth literacy and internet use only included the 3 education categories of secondary school or less, certificate or diploma, and university or higher [5,65]. Given that 17.9% (94/525) of the participants did not complete secondary school in this study (Table 1), it was decided that 4 categories of education would be appropriate. On the basis of existing studies, ICT use generally refers to access to, and use of, digital devices and the internet [43,47,60]. Therefore, ICT use was assessed by 3 survey questions, including number of digital devices used (range 0-4), number of ICT platforms used (range 0-10), and whether the participant had looked for web-based information in the last 3 months (yes or no). The number of digital devices used was determined by the question “Do you use any of the following devices?” with the answers including computer or laptop, mobile phone or smartphone, tablet, and other. The number of ICT platforms used was calculated by the participants’ answer to the question “Do you use any of the following to connect with others?” with the answers including email, text message, Facebook, Twitter, Instagram, Snapchat, WhatsApp or WeChat, blogging, forum or chat room, and other.

**Table 1:** Participant characteristics (N=525).

Characteristics		Value
Age (years), mean (SD; range)		56.8 (18.6; 18-94)
**Sex, n (%)**		
	Female	320 (61)
	Male	203 (38.7)
**Education, n (%)**		
	Less than secondary school	94 (17.9)
	Completed secondary school	106 (20.2)
	Certificate or diploma	141 (26.9)
	Completed university or higher	175 (33.3)
Spoke English at home, n (%)		363 (69.1)
**Ownership of digital device (a person may have more than one device), n (%)**		
	Computer or laptop	372 (71.2)
	Mobile phone or smartphone	459 (87.4)
	Tablet	241 (45.9)
	Other	6 (1.1)
Average number of digital devices owned, mean (SD; range)		2.1 (0.9; 0-4)
**Use of digital communication platform (a person may use more than one platform), n (%)**		
	Email	394 (75)
	Text message	398 (75.8)
	Facebook	266 (50.7)
	Twitter	30 (5.7)
	Instagram	104 (19.8)
	Snapchat	51 (9.7)
	WhatsApp or WeChat	112 (21.3)
	Blogging	15 (2.9)
	Forum or chat room	26 (5)
	Other	9 (1.7)
Average number of digital platforms used, mean (SD; range)		2.7 (1.8; 0-10)
Looked for web-based information in the last 3 months, n (%)		392 (74.4)

### Statistical Analysis

To evaluate known-groups validity, an important prerequisite for hypothesis testing is evidence of measurement equivalence or invariance across the groups [66], which refers to the stability of measurement across the different groups [67,68]. Measurement nonequivalence can occur when the characteristics of certain groups or grouping variables that are irrelevant to the construct being measured affect how people respond to the measurement [68]. Hence, group differences cannot be satisfactorily established if measurement invariance across group is not examined [69,70].

To evaluate measurement invariance, DIF is a common statistical observation that signals whether an item is functioning differentially across the grouping variables [71,72]. The presence of DIF indicates that there is a direct effect from a grouping variable on an item net of the association between the grouping variable and the latent construct. As such, the item is not measuring what it is intended to measure, and the estimated group differences are biased [68,73]. There are 2 types of DIF: uniform DIF and nonuniform DIF. Uniform DIF occurs when a group scores consistently and systematically higher or lower on a specific item than the other groups across all levels of ability, whereas nonuniform DIF is detected when the probability of endorsing an item among the groups varies across different ability levels [68,72,74]. According to the *Standards*, the main concern is uniform DIF because it can lead to “systematically different responses to a particular item” [27].

To ensure that DIF was considered, analysis using the multiple indicators multiple causes (MIMIC) model approach was chosen for this study. The MIMIC model is a type of structural equation modeling (SEM), which contains a *measurement model* that describes the relationship of the latent variables and their observed variables and a *structural model* that describes the links among the latent variables [75]. Therefore, taking the SEM approach to known-groups analysis with latent variables will account for measurement error in the outcome variables [76]. This approach also “allows simultaneous factor analysis and regression of factor scores on covariates for the comparison of item functioning across groups, while accounting for differences in several personal characteristics” [77]. In a MIMIC model, covariates (represented by group membership) can be categorical or continuous such that continuous variables (eg, age) do not have to be divided into arbitrary groups as in other statistical methods [77,78]. The MIMIC model approach has also been shown to have superior performance even with small or uneven group sample sizes compared with using other approaches [70,74,79]. As the DIF and known-groups analysis for this study involved both categorical and continuous variables, and small and uneven sample sizes for certain groups existed, the MIMIC model was considered suitable. Although the MIMIC model approach only tests for uniform DIF, rather than nonuniform DIF, the method was still considered appropriate because uniform DIF is more likely to occur than nonuniform DIF [80], and the main purpose of collecting DIF evidence, according to the *Standards*, is to identify systematically different responses [27].

Furthermore, a Bayesian approach was used for the MIMIC model in this study. It has been argued that the Bayesian approach is a better reflection of substantive theories because it is less restrictive; in addition, it does not rely on data with normal distribution and performs well with a small sample size [80,81]. A few studies have pointed to several advantages of using Bayesian SEM modeling over the traditional *frequentist* approaches such as maximum likelihood [80,81]. Of particular relevance to this study, these studies have highlighted the benefit that Bayesian SEM offers for investigating covariances among item residuals and potential cross-loadings that can be hypothesized to be *approximately* zero rather than exactly zero as in traditional SEM [82]. The same strategy was used here by including uniform DIF in the model.

For a Bayesian MIMIC model, informative small variance priors are applied to the DIF paths. A prior variance of 0.01 or 0.02 means that 95% of the variation lies within the ranges of ±0.20 or ±0.28 [81,82]. In addition, informative priors are also given to the residual covariances using the inverse-Wishart distribution, “a standard prior distribution for covariance matrices in Bayesian analysis” [81]. The application involves testing a model with a large enough *df* of the inverse-Wishart distribution and gradually lowering the *df* parameter to find a more flexible model [82]. As such, several models with different informative priors are usually tested and compared to identify the model of interest, which is the model that is not rejected by the data and can be considered closest to the frequentist model that fits well enough [82]. Model fit in the Bayesian approach is evaluated by the posterior predictive P value (*PPP*) and 95% CI for the difference between the observed and replicated chi-square values. *PPP*<.05 and positive 95% CI indicate a misfit, and *PPP* of approximately .50 and a value of zero falling close to the middle of the 95% CI indicates an excellent fit [81]. Models can further be compared by examining model convergence and discrepancy information criterion with quicker convergence (potential scale reduction being consistently less than 1.05) and lower discrepancy information criterion value to be chosen as the model of interest [82].

For the MIMIC model, age, sex, education, ICT use, and language were included as covariates. However, given that research indicated that age, sex, and education were also associated with ICT use and skills [31,32,43], ICT use was also tested as a mediator. As such, a mediated MIMIC model—a model that allows for identifying DIF and group differences as well as providing insights into the underlying mechanism [83]—was set up for this study.

**Figure 1:**
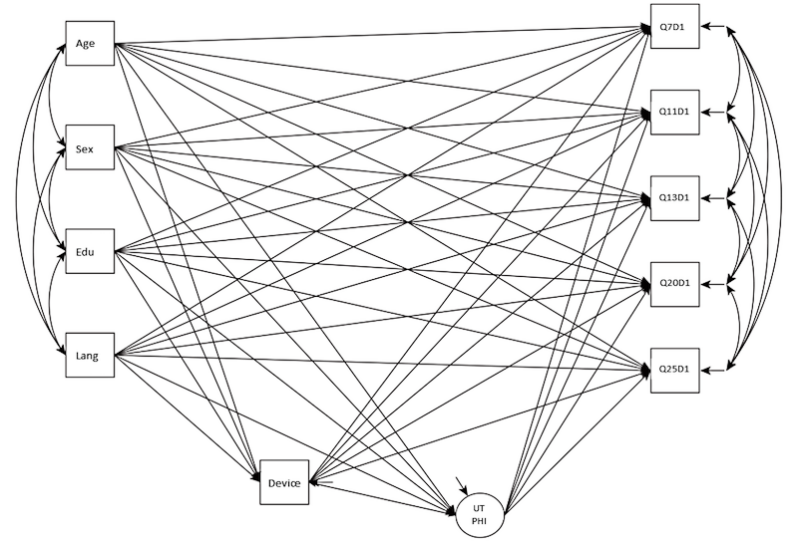
Bayesian multiple indicators multiple causes model for known-groups validity and differential item functioning testing with scale 1 of the eHealth Literacy Questionnaire as an example. Output from Mplus [87]: Age: range 18-94 years; Device: information and communication technology use represented by the number of devices used (range 0-4); Edu: Education: 1=less than secondary school, 2=completed secondary school, 3=certificate or diploma, and 4=completed university or higher; Lang: Language spoken at home: 0=English and 1=other languages; Q7D1, Q11D1, Q13D1, Q20D1, and Q25D1: eHealth Literacy Questionnaire items; Sex: 0=male and 1=female; UTPHI: eHealth Literacy Questionnaire scale 1: Using technology to process health information.

Given that there were 3 indicators (digital devices, ICT platforms, and search for web-based information) for ICT use, a final setup for the MIMIC model was to identify the single best indicator for ICT use. It has been argued that the single best indicator is sufficient for developing theoretically sophisticated models [84]. This was determined by running a Bayesian one-factor confirmatory factor analysis model of ICT use, with no prior, using the 3 indicators to find the one with the highest factor loading to represent ICT use. The result indicated an excellent model fit (*PPP*=.49, 95% CI for the difference between observed and replicated chi-square values –11.81 to 12.25), and number of devices was identified with the highest loading (0.81) on ICT use (Multimedia Appendix 1). Hence, *device* was used to represent ICT use in the final MIMIC model. The mediated DIF model using scale 1 of the eHLQ as an example is shown in Figure 1. As there is no consensus on sample size for Bayesian MIMIC model testing, which may range from 300 to more than 500 [79,85], a minimum sample size of 500 was estimated.

Descriptive statistics were conducted using SPSS, version 25.0 (IBM Corporation) [86], and the Bayesian MIMIC model testing was run using Mplus software version 8.3 [87]. For model selection, a sequence of one-factor models for each of the 7 factors was fitted to the data by varying the informative priors for *df* of the inverse-Wishart distribution=200, 150, 100, 80, and 60. The results would inform the use of the prior for residual covariance for the MIMIC model testing. Next, 3 models using the chosen prior combined with informative priors for DIF paths (variance=0.01, 0.015, and 0.02) were fitted to the data. Model estimation was performed with 50,000 iterations.

To determine the group differences, direct effect, indirect effect, and total effect produced in the Mplus outputs were examined. A significant total effect indicated significant group differences. A significant direct effect indicated that group differences existed independent of any mediating effect, whereas a significant indirect effect indicated that group differences were mediated through ICT use. Mplus produced the results of one-tailed P values and indicated that P<.025 was significant. However, because the hypotheses for age, sex, education, and ICT use were directional, P<.05 was considered significant, whereas P<.025 remained significant for either a positive or negative effect for language because no hypothesis was set a priori. A further calculation of mediation proportion was undertaken to gain deeper understanding of the extent of mediation. Mediation proportion refers to the portion of effect on an outcome explained by an intermediate variable. It is calculated by dividing the indirect effect by the total effect [88]. It should be noted that computation of mediation proportion is considered not appropriate if the total effect is too small, that is, less than ±0.2, or in case of inconsistent mediation, that is, opposite signs for the estimates. If mediation proportion is at least 0.8, complete mediation can be claimed. As it was cautioned that statistical significance was sensitive to sample size and the effects should not be evaluated simply based on statistical significance, the significant group differences were further investigated by examining the size of the effect estimates to determine if the effects were practically significant or could be ignored because the size of the effect would have no appreciable bearing on the interpretation of group differences [89].

For the evaluation of DIF, a significant direct effect from the covariate on the observed variable, that is, the questionnaire item, indicated the presence of DIF [90-92], and one-tailed P<.025 was considered significant because no directional hypotheses were set up for DIF. If DIF was identified, the prior-posterior predictive P value (*PPPP*) from the model of interest needed to be examined. The *PPPP* is a value used “for the evaluation of hypotheses specifying small variance priors for the parameters of interest” [93]. It is about whether the informative priors for DIF can be considered approximate zero. If *PPPP*>.05, that is, it can be considered nonsignificant, the estimates of the DIF are considered approximate zero and are thus ignorable. Hence, if the model of interest has a variance prior of 0.01 and the *PPPP* is nonsignificant, then estimates within the range of ±0.20 could be considered ignorable [94].

## Results

### Participant Characteristics

A total of 525 responses were included for analysis. The mean age of the participants was 56.8 (SD 18.6) years. Of the 525 participants, 320 (61%) were women, 175 (33.3%) had a university education, and 162 (30.9%) spoke a language other than English at home. Ownership of digital devices was generally high, but of the 525 participants, 66 (12.6%) did not have a mobile phone and 153 (28.8%) did not have a computer or laptop, whereas 133 (25.6%) did not search for any web-based information (see Table 1 for participant characteristics). The scale scores are shown in Table 2. The results showed that the participants seemed to have relatively good knowledge about their health conditions (scale *2. Understanding of health concepts and language*: mean 2.95, SD 0.41), but they might not always use technology for health (*1. Using technology to process health information*: mean 2.59, SD 0.61 and *5. Motivated to engage with digital services*: mean 2.63, SD 0.55). Although the participants were generally comfortable with the privacy and security of digital health systems (*4. Feel safe and in control*: mean 2.83, SD 0.49), they were less likely to consider that the systems met their individual needs (*7. Digital services that suit individual needs*: mean 2.43, SD 0.57).

**Table 2:** eHealth Literacy Questionnaire scale scores (N=525; score range 1-4).

Scale	Value, mean (SD)	Missing data
1. Using technology to process health information	2.59 (0.61)	0
2. Understanding of health concepts and language	2.95 (0.41)	0
3. Ability to actively engage with digital services	2.65 (0.68)	1
4. Feel safe and in control	2.83 (0.49)	5
5. Motivated to engage with digital services	2.63 (0.55)	0
6. Access to digital services that work	2.64 (0.45)	1
7. Digital services that suit individual needs	2.43 (0.57)	11

### DIF Influence

Modeling testing identified *df*=60 as the prior for residual covariance, with subsequent testing of the 3 models all achieving good fit with similar results (Multimedia Appendix 2). Hence, the most restrictive model with prior variance for DIF path of 0.01 was chosen as the model of interest. The *PPP*s of the 7 scales ranged from .32 to .38, and all *PPPP*s were nonsignificant.

With the selected model of interest, significant direct effects were found for 2 items indicating possible DIF. However, both estimates were within the range of ±0.2; therefore, they were considered ignorable [94] (Multimedia Appendix 3). Thus, the results indicated no or ignorable DIF influence of ICT use, age, sex, education, and language on the scores of the 35 eHLQ items.

### Known-Groups Validity

#### Mixed Evidence

The evidence on the relations of eHLQ scores to other variables based on known-groups validity is mixed, with some of the hypotheses supported (see Table 3 for estimated effects).

**Table 3:** Estimated effects of age, sex, education, language, and information and communication technology (ICT) use (device) on the 7 eHealth literacy latent variables.

eHealth literacy latent variable and eHealth Literacy Questionnaire scale			Total effect^a^		Direct effect^a^		Indirect effect^a^		Mediation proportion
**Age^b^**									
	1. Using technology to process health information	–*0.32 (0.08)*^c,d^		–*0.22 (0.08)*		–*0.10 (0.02)*		0.31	
	2. Understanding of health concepts and language	–0.05 (0.12)		–0.01 (0.12)		–0.05 (0.03)		N/A^e^	
	3. Ability to actively engage with digital services	–*0.37 (0.07)*		–*0.26 (0.07)*		–*0.12 (0.02)*		0.32	
	4. Feel safe and in control	–0.01 (0.10)		0.01 (0.10)		–0.02 (0.03)		N/A	
	5. Motivated to engage with digital services	–*0.21 (0.09)*		–0.13 (0.09)		–*0.08 (0.03)*		0.38	
	6. Access to digital services that work	–0.08 (0.10)		–0.03 (0.10)		–*0.05 (0.03)*		N/A	
	7. Digital services that suit individual needs	–*0.21 (0.09)*		–0.13 (0.10)		–*0.08 (0.03)*		0.38	
**Sex^f^**									
	1. Using technology to process health information	–0.04 (0.05)		–0.04 (0.05)		–0.01 (0.02)		N/A	
	2. Understanding of health concepts and language	0.01 (0.07)		0.01 (0.07)		–0.00 (0.01)		N/A	
	3. Ability to actively engage with digital services	–0.06 (0.05)		–0.05 (0.05)		–0.01 (0.02)		N/A	
	4. Feel safe and in control	0.04 (0.06)		0.04 (0.06)		0.00 (0.01)		N/A	
	5. Motivated to engage with digital services	–*0.12 (0.06)*		–*0.11 (0.06)*		–0.00 (0.01)		N/A	
	6. Access to digital services that work	–0.01 (0.06)		–0.08 (0.06)		–0.00 (0.01)		N/A	
	7. Digital services that suit individual needs	–0.09 (0.06)		–0.09 (0.06)		–0.00 (0.01)		N/A	
**Education^g^**									
	1. Using technology to process health information	*0.22 (0.09)*		0.09 (0.09)		*0.13 (0.03)*		0.59	
	2. Understanding of health concepts and language	0.18 (0.13)		0.12 (0.13)		0.06 (0.04)		N/A	
	3. Ability to actively engage with digital services	*0.25 (0.08)*		0.11 (0.08)		*0.14 (0.03)*		0.56	
	4. Feel safe and in control	–0.03 (0.11)		–0.06 (0.11)		0.03 (0.03)		N/A	
	5. Motivated to engage with digital services	0.12 (0.10)		0.02 (0.10)		*0.11 (0.03)*		N/A	
	6. Access to digital services that work	–0.04 (0.11)		–0.11 (0.11)		*0.07 (0.03)*		N/A	
	7. Digital services that suit individual needs	0.11 (0.10)		0.01 (0.10)		*0.10 (0.03)*		N/A	
**Language^h^**									
	1. Using technology to process health information	–0.02 (0.05)		0.03 (0.05)		–*0.06 (0.02)*		N/A	
	2. Understanding of health concepts and language	–*0.15 (0.07)*		–0.12 (0.07)		–0.03 (0.02)		N/A	
	3. Ability to actively engage with digital services	–0.09 (0.05)		–0.03 (0.05)		–*0.07 (0.02)*		N/A	
	4. Feel safe and in control	–0.09 (0.06)		–0.08 (0.06)		–0.01 (0.02)		N/A	
	5. Motivated to engage with digital services	–0.01 (0.06)		0.04 (0.06)		–*0.05 (0.02)*		N/A	
	6. Access to digital services that work	–0.02 (0.06)		0.02 (0.06)		–*0.03 (0.02)*		N/A	
	7. Digital services that suit individual needs	–0.03 (0.06)		0.02 (0.06)		–*0.04 (0.02)*		N/A	
**ICT use (device)^i^**									
	1. Using technology to process health information	N/A		*0.38 (0.07)*		N/A		N/A	
	2. Understanding of health concepts and language	N/A		0.18 (0.11)		N/A		N/A	
	3. Ability to actively engage with digital services	N/A		*0.42(0.06)*		N/A		N/A	
	4. Feel safe and in control	N/A		0.09 (0.10)		N/A		N/A	
	5. Motivated to engage with digital services	N/A		*0.31 (0.08)*		N/A		N/A	
	6. Access to digital services that work	N/A		*0.20 (0.09)*		N/A		N/A	
	7. Digital services that suit individual needs	N/A		*0.30 (0.08)*		N/A		N/A	

^a^Standardized estimates reported.

^b^Age: range 18-94 years.

^c^Posterior SD for estimates shown in parentheses.

^d^Italicized values indicated statistically significant differences with P<.05 for information and communication technology use (device), age, sex, and education and P<.025 for language.

^e^N/A: not applicable. For age, sex, education and language, *not applicable* is due to inconsistent mediation, total effect less than ±0.2, or lack of indirect effect [89]; for information and communication technology use (device), *not applicable* is due to the fact that it is treated as the mediator.

^f^Sex code: 0=male and 1=female.

^g^Education code: 1=less than secondary school, 2=completed secondary school, 3=certificate or diploma, and 4=completed university or higher.

^h^Language code: 0=spoke English at home and 1=spoke other language at home.

^i^Information and communication technology use (device): number of devices used, range 0-4.

#### Age

H1: Age is negatively related to the scores on all latent variables representing the 7 scales.

This hypothesis was supported for only 4 of the expected latent variables. Being older was most strongly related to lower scores in *3. Ability to actively engage with digital services*, with a total effect of –0.37 (posterior SD 0.07; P<.001). Age also had quite a strong negative effect on *1. Using technology to process health information*, with a total effect of –0.32 (posterior SD 0.08; P<.001). A total effect of –0.21 (posterior SD 0.09) was found for both *5. Motivated to engage with digital services* and *7. Digital services that suit individual needs*, with P=.01 and P=.02, respectively. For all 4 latent variables with significant total effect, approximately two-thirds was a direct effect.

#### Sex

H2a: Being female is related to higher scores on the 3 latent variables representing the scales *1. Using technology to process health information*, *2. Understanding of health concepts and language*, and *5. Motivated to engage with digital services*.

H2b: Sex is not related to score differences on the 4 latent variables representing the other 4 scales.

H2a was not supported, whereas H2b was supported. Sex was not related to the score differences in any of the latent variables. Although a significant total effect was found for the scale *5. Motivated to engage with digital services*, the estimate was –0.12, which was less than ±0.2 and was considered a *too small* effect [89] and therefore ignorable. It was also noted that the mediating effect of ICT use was 0 or close to 0 for all 7 latent variables.

#### Education

H3: Education is positively related to the scores on all latent variables representing the 7 scales.

This hypothesis was supported only for 2 of the 7 expected latent variables. Higher education was associated with higher scores in the latent variables representing *1. Using technology to process health information* (total effect 0.22, posterior SD 0.09; P=.01), with 59% of the effect mediated by ICT use, and *3. Ability to actively engage with digital services* (total effect 0.25, posterior SD 0.08; P<.001), with 56% of the effect mediated by ICT use.

#### ICT Use

H4: ICT use is positively related to the scores on the 6 latent variables representing scales 1, 3, 4, 5, 6, and 7 but not to the score on the latent variable representing *2. Understanding of health concepts and language*.

This hypothesis was supported except for the latent variable representing *4. Feel safe and in control*. Higher ICT use had the strongest relation to higher scores in *3. Ability to actively engage with digital services*, with a direct effect of 0.42 (posterior SD 0.06; P<.001), followed by *1. Using technology to process health information*, with a direct effect of 0.38 (posterior SD 0.07; P<.001). The other latent variables with significant positive effect included *5. Motivated to engage with digital services* (direct effect 0.31, posterior SD 0.08; P<.001), *6. Access to digital services that work* (direct effect 0.20, posterior SD 0.09; P=.02), and *7. Digital services that suit individual needs* (direct effect 0.30, posterior SD 0.08; P<.001).

#### Language

No group differences were found between the participants who spoke English and those who spoke a language other than English at home. Although a significant total effect was found for the latent variable representing *2. Understanding of health concepts and language*, the effect size was ignorable because the estimate of –0.15 was considered too small [89].

## Discussion

### Principal Findings

This study used a Bayesian mediated MIMIC model approach to collect evidence based on relations to other variables to evaluate the eHLQ as a tool to understand eHealth literacy needs, using data collected from known-groups validity in the Australian community health context. Hypotheses for the expected score differences for age, sex, education, ICT use, and speaking English at home or not were supported for some of the eHealth literacy latent variables represented by the relevant eHLQ scales but not all. The results also confirmed measurement invariance across 5 demographic groups. This is important because the presence of measurement invariance indicates that when the eHLQ is applied to compare population groups with different demographic compositions in the Australian health setting, unbiased estimates of mean group differences are obtained, which provide reliable data to researchers, clinicians, and policy makers.

Although the *Standards*, the authoritative validity-testing reference, suggests that the expected score differences among groups can be an important source of evidence for validity [27], it was noted during hypotheses setting in this study that empirical findings on predictors of eHealth literacy are still limited and can be inconsistent. This is likely due to the lack of consensus on what is being measured and the use of a limited range of tools to measure the concept. Hence, the hypotheses for known-groups validity in this study need to be interpreted with caution. In contrast, this study is the first to explore group differences on eHealth literacy evaluated as a concept of 7 domains instead of simply focusing, as in other studies, on seeking or evaluating health information or using eHealth services.

This study is based on a contemporary statistical method, the Bayesian mediated MIMIC model, rather than methods frequently used in previous and current eHealth literacy research specifically and psychometric research in general. This could also be one of the reasons why the findings from this study are somewhat different from those of current studies. Common practice in testing for known-groups validity uses statistical methods such as the independent sample one-tailed or two-tailed *t* test or analysis of variance or relevant nonparametric tests to establish group differences. However, these methods do not take into account DIF as a potential confounding factor. Without establishing measurement invariance across demographic groups, any apparent group differences detected cannot be ascertained. Apart from being able to detect group differences as well as DIF in using a MIMIC model, this SEM approach accounts for measurement errors, and the use of the Bayesian approach also allows for an evaluation that is a better reflection of the real world, whereas the inclusion of a mediator adds valuable information to the underlying mechanism of the group differences detected. Unlike *t* tests or analysis of variance, age was not divided into arbitrary groups but was treated as a continuous variable. Therefore, the results of this study, in fact, provide new and unbiased insights into the predictors of eHealth literacy.

The findings in this study indicated that being older had lower scores in 4 scales, with *3. Ability to actively engage with digital services* having the strongest effect, followed by *1. Using technology to process health information*, *5. Motivated to engage with digital services*, and *7. Digital services that suit individual needs*. This result is generally consistent with existing studies. However, an interesting result from the mediated MIMIC model is that most of the effect of age was not mediated by ICT use, indicating that ICT use may have a limited role in these 4 domains of eHealth literacy. A possible factor is the cognitive, motor, and sensory decline associated with aging, as frequently suggested in the literature [35-42]. Such findings may also imply that simply providing ICT training may not be adequate with regard to enhancing eHealth literacy among older people, and other interventions are necessary. The focus on computer skills and ICT training as the main mechanism of current eHealth literacy interventions [95] not only may not be effective for older people, but other domains of eHealth literacy are also likely to be overlooked.

Another result of interest is the relationship between education and the eHealth literacy domains because education is only positively associated with the scales *1. Using technology to process health information* and *3. Ability to actively engage with digital services*. Unlike in the case of age, most of the effects were mediated by ICT use, confirming the role of ICT use in enhancing certain aspects of eHealth literacy. As such, providing ICT education and training is likely to benefit people with lower education in increasing their ability to engage with digital services and enhancing use of technology for health. Such a finding suggests that other efforts are required to address the eHealth literacy needs concerning privacy, motivation, or access to suitable digital services. In contrast, why education was not related to *2. Understanding of health concepts and language* in the digital context warrants further investigation.

Sex having no relationship with any of the eHLQ scale scores suggests that sex may not be a good predictor of eHealth literacy. This may be due to the narrowing gap in education between the sexes in recent years and the fact that technology use has become an indispensable part of modern-day life for most people. Although the hypothesis of ICT use is mostly supported, the results show that ICT use is not associated with the eHealth literacy domain of feeling safe and in control. This again reiterates that technical skills in ICT training are inadequate to address all eHealth literacy needs. These findings also call for assessment of eHealth literacy using unbiased multidimensional questionnaires such as the eHLQ so that eHealth literacy needs can be clearly identified and addressed.

Although the aim of this study is to collect evidence based on relations to other variables, the statistical method used also established the robustness of the internal structure of the eHLQ in terms of measurement invariance across 5 demographic groups. The presence of DIF indicates that a questionnaire item is not measuring what is intended, and thus the resulting scores may be biased [68]. Given the issue of health disparities among different sociodemographic groups, the presence of DIF in patient-reported outcome measures may lead to inaccurate interpretation of scores and inappropriate health care decisions [96]. Hence, providing clear evidence of measurement invariance across the 5 demographic groups is an important finding. The Danish validity testing also found no evidence of influence of age and sex on the item scores in the Danish setting [21]. With the accumulating evidence on DIF, users of the eHLQ in similar Australian and Danish contexts can be assured that the mean scores obtained from the eHLQ can be interpreted properly to address the different needs of different groups. As such, the validity evidence collected in this study supports the eHLQ as a tool to understand eHealth literacy needs and helps to inform the development of fit-for-purpose health interventions [26].

### Limitations

A limitation of this study is that the hypotheses were based on limited empirical findings of eHealth literacy predictors such that the results may need to be interpreted differently. Although the MIMIC model approach has the advantage of evaluating both group differences and DIF simultaneously, only uniform DIF was tested, and nonuniform DIF was not investigated. Nevertheless, uniform DIF is a more important threat to validity because it can lead to systematic score differences on account of group characteristics [27]. In examining evidence pertaining to relations to other variables, this study only focused on data collected from known-groups validity. Although the relationship with other instruments could not be tested because of the lack of an instrument to measure the same constructs, whether the test scores can be generalized to other settings or contexts is another potential source of evidence on relations to other variables [27] that warrants investigation in future studies. Because of limited resources and because the health sites were always busy during the data collection period, it was difficult to gauge the number of people coming through the door, and no response rate was recorded. However, the participants’ characteristics demonstrated a generally well-represented sample. Unlike the Danish validity testing, which included the general population in various community settings, this study only focused on the community health setting, but data were nevertheless collected from different geographic locations, including both metropolitan and regional areas. Further testing of the eHLQ in other settings and cultures is required for the accumulation of validity evidence for the eHLQ.

### Conclusions

With health resources and services increasingly delivered through digital technologies, eHealth literacy has become an essential capability in the digital age. This study provides robust validity evidence of the eHLQ in the Australian community health setting. The evidence demonstrates that the tool can be used by health care providers and policy makers to gain unbiased and valuable insights into people’s diverse eHealth literacy needs so that tailored health interventions can be effectively developed in similar settings. The eHLQ can also be used to align the demand of any eHealth system with the eHealth literacy needs of users to optimize access and use of digital health among users and promote health equity.

## Appendix

Multimedia Appendix 1 Bayesian structural equation modelling for information and communication technology use.

Multimedia Appendix 2 Bayesian model fit information of the eHealth Literacy Questionnaire for the testing of known-groups validity and differential item functioning of age, sex, education, language, and information and communication technology use (device), with model 1 as the model of interest.

Multimedia Appendix 3 Estimates for the direct effect of eHealth Literacy Questionnaire items on information and communication technology use (device), age, sex, education, and language.

## Abbreviations

DIF: differential item functioning
eHEALS: eHealth Literacy Scale
eHLQ: eHealth Literacy Questionnaire
ICT: information and communication technology
MIMIC: multiple indicators multiple causes
PPP: posterior predictive P value
PPPP: prior-posterior predictive P value
SEM: structural equation modeling
