# Supplementary material for: Validity Evidence Based on Relations to Other Variables of the eHealth Literacy Questionnaire (eHLQ): Bayesian Approach to Test for Known-Groups Validity
Source: J Med Internet Res. 2021 Oct 14;23(10):e30243. doi: 10.2196/30243 (PMC8554672; doi:10.2196/30243)
Supplement: Multimedia Appendix 1 [file jmir_v23i10e30243_app1.doc]

**Multimedia Appendix 1:** Bayesian structural equation modelling for information and communication technology use.


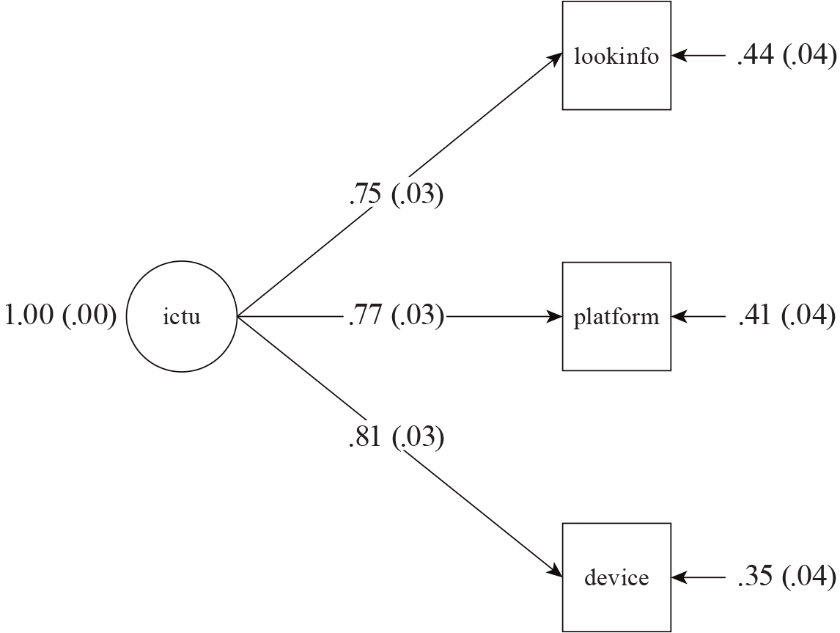


Output from Mplus.

ictu=information and communication technology use.

lookinfo=look for online information (0=no, 1=yes).

platform=number of platforms used (range=0–10).

device=number of devices used (range=0–4).
