# Supplementary material for: Validity Evidence Based on Relations to Other Variables of the eHealth Literacy Questionnaire (eHLQ): Bayesian Approach to Test for Known-Groups Validity
Source: J Med Internet Res. 2021 Oct 14;23(10):e30243. doi: 10.2196/30243 (PMC8554672; doi:10.2196/30243)
Supplement: Multimedia Appendix 2 [file jmir_v23i10e30243_app2.doc]

**Multimedia Appendix 2:** Bayesian model fit information of the eHealth Literacy Questionnaire for the testing of known-groups validity and differential item functioning of age, sex, education, language, and information and communication technology use (device), with model 1 as the model of interest.

| Model parameter | Model 1  0.01/60^a^ | Model 2  0.015/60^a^ | Model 3  0.02/60^a^ |
| --- | --- | --- | --- |
| **1. Using technology to process health information** | | | |
| PPP | **0.35** | 0.34 | 0.33 |
| 95% CI for *X*^2^ difference | **-24.41 – 35.81** | -24.18 – 36.11 | -23.90 – 36.34 |
| PPPP | **0.90** | 0.88 | 0.85 |
| DIC | **5,449.21** | 5,450.82 | 5,450.58 |
| N iterations to PSR consistently<1.05 | **4,900** | 18,800 | 19,400 |
| **2. Understanding of health concepts and language** | | | |
| PPP | **0.32** | 0.33 | 0.33 |
| 95% CI for *X*^2^ difference | **-23.25 – 36.90** | -23.62 – 36.64 | -23.71 – 36.53 |
| PPPP | **0.86** | 0.83 | 0.80 |
| DIC | **5,040.00** | 5,040.30 | 5,040.55 |
| N iterations to PSR consistently<1.05 | **4,600** | 19,100 | 22,300 |
| **3. Ability to actively engage with digital service** | | | |
| PPP | **0.34** | 0.34 | 0.33 |
| 95% CI for *X*^2^ difference | **-23.72 – 35.51** | -23.81 – 35.80 | -23.85 – 35.96 |
| PPPP | **0.89** | 0.89 | 0.86 |
| DIC | **5,446.39** | 5,447.19 | 5,447.67 |
| N iterations to PSR consistently<1.05 | **5,000** | 18,800 | 19,600 |
| **4. Feel safe and in control** | | | |
| PPP | **0.35** | 0.34 | 0.34 |
| 95% CI for *X*^2^ difference | **-24.62 – 35.59** | -24.51 – 35.81 | -24.47 – 36.02 |
| PPPP | **0.93** | 0.88 | 0.85 |
| DIC | **4,955.76** | 4,956.54 | 4,956.98 |
| N iterations to PSR consistently<1.05 | **5,100** | 6,800 | 18,800 |
| **5. Motivated to engage with digital services** | | | |
| PPP | **0.35** | 0.34 | 0.34 |
| 95% CI for *X*^2^ difference | **-24.29 – 35.48** | -24.10 – 35.81 | -24.07 – 36.10 |
| PPPP | **0.91** | 0.88 | 0.84 |
| DIC | **5,237.01** | 5,237.80 | 5,238.26 |
| N iterations to PSR consistently<1.05 | **4,900** | 18,800 | 19,400 |
| **6. Access to digital services that work** | | | |
| PPP | **0.34** | 0.32 | 0.32 |
| 95% CI for *X*^2^ difference | **-26.37 – 41.02** | -25.95 – 41.44 | -25.85 – 41.63 |
| PPPP | **0.79** | 0.79 | 0.79 |
| DIC | **6,326.19** | 6,327.41 | 6,328.12 |
| N iterations to PSR consistently<1.05 | **8,600** | 14,600 | 14,500 |
| **Model parameter** | **Model 1**  **0.01/60^a^** | **Model 2**  **0.015/60^a^** | **Model 3**  **0.02/60^a^** |
| **7. Digital services that suit individual needs** | | | |
| PPP | **0.38** | 0.38 | 0.37 |
| 95% CI for *X*^2^ difference | **-22.48 – 30.48** | -22.27 – 30.84 | -22.32 – 31.01 |
| PPPP | **0.90** | 0.86 | 0.81 |
| DIC | **4,130.79** | 4,131.36 | 4,131.68 |
| N iterations to PSR consistently<1.05 | **7,100** | 8,500 | 26,100 |
| ^a^informative prior variance for DIF paths/inverse-Wishart degrees of freedom for residual covariance.  PPP=Posterior predictive *P* value.  95% CI for X^2^ difference=95% confidence interval for the difference between observed and replicated Chi-square values.  PPPP=Prior-posterior predictive *P* value.  DIC = Discrepancy information criterion.  PSR=Potential scale reduction.  Bold=Model fit information of model of interest. | | | |
