# Supplementary material for: Validity Evidence Based on Relations to Other Variables of the eHealth Literacy Questionnaire (eHLQ): Bayesian Approach to Test for Known-Groups Validity
Source: J Med Internet Res. 2021 Oct 14;23(10):e30243. doi: 10.2196/30243 (PMC8554672; doi:10.2196/30243)
Supplement: Multimedia Appendix 3 [file jmir_v23i10e30243_app3.doc]

**Multimedia Appendix 3:** Estimates for the direct effect of eHealth Literacy Questionnaire items on information and communication technology use (device), age, sex, education, and language.

| Scale/Item | | Device | Age | Sex | Education | Language |
| --- | --- | --- | --- | --- | --- | --- |
| **1. Using technology to process health information** | | | | | | |
| Q7D1 | I use technology to find… | 0.07 (0.06) | -0.08 (0.06) | 0.02 (0.04) | 0.06 (0.07) | -0.01 (0.03) |
| Q11D1 | I often use technology… | 0.05 (0.06) | -0.03 (0.07) | 0.02 (0.04) | 0.02 (0.07) | -0.03 (0.04) |
| Q13D1 | Technology helps me… | -0.09 (0.07) | 0.03 (0.07) | 0.02 (0.04) | -0.04 (0.08) | 0.00 (0.04) |
| Q20D1 | I use technology to share… | -0.01 (0.07) | 0.10 (0.07) | -0.03 (0.04) | -0.02 (0.07) | 0.04 (0.04) |
| Q25D1 | I use technology to organise… | -0.04 (0.07) | 0.01 (0.07) | -0.03 (0.04) | -0.04 (0.07) | 0.02 (0.04) |
| **2. Understanding of health concepts and language** | | | | | | |
| Q5D2 | The knowledge I have helps me… | 0.07 (0.08) | -0.02 (0.08) | 0.03 (0.05) | -0.02 (0.09) | 0.03 (0.05) |
| Q12D2 | I have enough information… | 0.02 (0.08) | -0.00 (0.08) | 0.09 (0.05) | -0.01 (0.09) | **-0.10 (0.05)** |
| Q15D2 | I understand medical results… | -0.06 (0.08) | 0.03 (0.08) | -0.04 (0.05) | 0.07 (0.09) | 0.03 (0.05) |
| Q21D2 | Overall, I understand how… | -0.08 (0.08) | -0.03 (0.09) | 0.04 (0.05) | 0.00 (0.10) | -0.08 (0.05) |
| Q26D2 | I use measurements about my body… | 0.03 (0.08) | 0.04 (0.08) | -0.09 (0.05) | -0.01 (0.09) | 0.09 (0.05) |
| **3. Ability to actively engage with digital services** | | | | | | |
| Q4D3 | I know how to use technology to get… | -0.01 (0.07) | 0.08 (0.07) | 0.03 (0.04) | -0.10 (0.07) | 0.01 (0.04) |
| Q6D3 | I know how to make technology… | 0.03 (0.06) | -0.02 (0.06) | -0.00 (0.03) | -0.03 (0.07) | 0.00 (0.03) |
| Q8D3 | I can enter data into health… | -0.01 (0.06) | 0.07 (0.06) | 0.05 (0.04) | 0.05 (0.07) | -0.02 (0.03) |
| Q17D3 | I quickly learn how to find my way… | -0.01 (0.06) | -0.11 (0.06) | -0.03 (0.03) | 0.06 (0.06) | -0.02 (0.03) |
| Q32D3 | I easily learn to use new… | -0.00 (0.06) | -0.02 (0.06) | -0.05 (0.04) | 0.04 (0.07) | 0.03 (0.03) |
| **4. Feel safe and in control** | | | | | | |
| Q1D4 | I am sure that my health data… | -0.01 (0.07) | -0.06 (0.08) | 0.04 (0.04) | 0.00 (0.08) | -0.03 (0.04) |
| Q10D4 | My electronic healthcare data… | 0.03 (0.07) | -0.04 (0.08) | -0.03 (0.04) | -0.02 (0.09) | -0.04 (0.04) |
| Q14D4 | I have a clear understanding… | 0.04 (0.07) | **0.14 (0.07)** | -0.00 (0.04) | 0.04 (0.07) | 0.04 (0.04) |
| Q22D4 | I am sure that only authorised people… | -0.08 (0.08) | -0.04 (0.08) | -0.02 (0.04) | -0.01 (0.09) | 0.02 (0.04) |
| Q30D4 | I am confident that healthcare providers… | 0.05 (0.07) | 0.02 (0.08) | 0.02 (0.04) | -0.03 (0.08) | 0.00 (0.04) |
| **5. Motivated to engage with digital services** | | | | | | |
| Q2D5 | Technology makes me feel actively… | 0.05 (0.07) | 0.02 (0.07) | 0.04 (0.04) | -0.11 (0.08) | -0.02 (0.04) |
| Q19D5 | I find technology helps me to… | 0.02 (0.07) | -0.01 (0.07) | 0.01 (0.04) | 0.07 (0.08) | -0.05 (0.04) |
| Q24D5 | I find I get better services… | -0.08 (0.07) | 0.03 (0.07) | -0.00 (0.04) | 0.01 (0.08) | 0.05 (0.04) |
| Q27D5 | Technology improves my communication… | -0.03 (0.07) | 0.02 (0.07) | 0.01 (0.04) | 0.03 (0.08) | 0.02 (0.04) |
| Q35D5 | I find technology useful for monitoring… | 0.04 (0.07) | -0.05 (0.07) | -0.06 (0.04) | -0.00 (0.08) | 0.00 (0.04) |
| **6. Access to digital services that work** | | | | | | |
| Q3D6 | Information about my health is… | -0.09 (0.06) | 0.10 (0.07) | 0.01 (0.04) | -0.09 (0.07) | -0.04 (0.04) |
| Q9D6 | My healthcare providers deliver services… | 0.10 (0.07) | -0.11 (0.07) | 0.01 (0.04) | 0.02 (0.08) | 0.02 (0.04) |
| Q16D6 | My health data are available… | -0.11 (0.07) | 0.12 (0.08) | 0.03 (0.04) | -0.04 (0.08) | -0.01 (0.04) |
| Q23D6 | All the health technology… | -0.03 (0.07) | 0.03 (0.08) | -0.01 (0.04) | 0.04 (0.09) | 0.04 (0.04) |
| Q29D6 | Most of my healthcare providers can… | 0.05 (0.07) | -0.08 (0.07) | -0.01 (0.04) | -0.03 (0.07) | 0.01 (0.04) |
| Q34D6 | I have access to health technology… | 0.13 (0.07) | -0.11 (0.07) | -0.04 (0.04) | 0.09 (0.08) | -0.03 (0.04) |
| **7. Digital services that suit individual needs** | | | | | | |
| Q18D7 | I find that eHealth systems adapt… | 0.04 (0.07) | -0.06 (0.08) | 0.01 (0.04) | -0.02 (0.08) | 0.01 (0.04) |
| Q28D7 | I find eHealth systems seem to… | -0.02 (0.07) | 0.05 (0.08) | -0.01 (0.04) | 0.00 (0.08) | 0.00 (0.04) |
| Q31D7 | I find eHealth systems are provided… | -0.04 (0.07) | 0.03 (0.08) | 0.02 (0.04) | -0.02 (0.08) | -0.04 (0.04) |
| Q33D7 | eHealth systems provide me… | 0.02 (0.07) | -0.02 (0.08) | -0.02 (0.04) | 0.04 (0.08) | 0.02 (0.04) |
| Items are truncated. Please contact the authors for full items.  Standardized estimates reported.  Posterior standard deviation for estimates shown in parentheses.  Bold=statistically significant differences, one-tailed significant if *P*<.025.  ICT Use/device=number of devices used range: 0–4; Age=range: 18–94; Sex code: 0=male, 1=female; Education code: 1=did not complete secondary school, 2=completed secondary school, 3=certificate/diploma, 4=completed university or above; Language code: 0=spoke English at home, 1=spoke other language at home. | | | | | | |
